# The dynamics of nasopharyngeal *streptococcus *pneumoniae carriage among rural Gambian mother-infant pairs

**DOI:** 10.1186/1471-2334-10-195

**Published:** 2010-07-05

**Authors:** Momodou K Darboe, Anthony JC Fulford, Ousman Secka, Andrew M Prentice

**Affiliations:** 1 MRC International Nutrition Group, Keneba Field Station, P. O. Box 273, Banjul The Gambia; 2 MRC Laboratories, Bacterial Disease Programme, Fajara, P. O. Box 273, Banjul The Gambia

## Abstract

**Background:**

*Streptococcus pneumoniae *is an important cause of community acquired pneumonia, sepsis, meningitis and otitis media globally and has been incriminated as a major cause of serious childhood bacterial infections in The Gambia. Better understanding of the dynamics of transmission and carriage will inform control strategies.

**Methods:**

This study was conducted among 196 mother-infant pairs recruited at birth from six villages in the West Kiang region of The Gambia. Nasopharyngeal swabs were collected from mother-infant pairs at birth (within 12 hours of delivery), 2, 5 and 12 months. Standard techniques of culture were used to identify carriage and serotype *S. pneumoniae*.

**Results:**

Of 46 serotypes identified, the 6 most common, 6A, 6B, 14, 15, 19F and 23F, accounted for 67.3% of the isolates from infants. Carriage of any serotype among infants rose from 1.5% at birth to plateau at approximately 80% by 2 m (prevalence at 2 m = 77%; 5 m = 86%; 12 m = 78%). Likewise, maternal carriage almost doubled in the first 2 months post-partum and remained elevated for the next 10 m (prevalence at birth = 13%; 2 m = 24%; 5 m = 22%; 12 m = 21%). Carriage was significantly seasonal in both infants and mothers with a peak in December and lowest transmission in August. The total number of different serotypes we isolated from each infant varied and less than would be expected had the serotypes assorted independently. In contrast, this variability was much as expected among mothers. The half-life of a serotype colony was estimated to be 1.90 m (CI_95%_: 1.66-2.21) in infants and 0.75 m (CI_95%_: 0.55-1.19) in mothers. While the odds for a serotype to be isolated from an infant increased by 9-fold if it had also been isolated from the mother, the population attributable fraction (PAF) of pneumococcal carriage in infants due to maternal carriage was only 9.5%. Some marked differences in dynamics were observed between vaccine and non-vaccine serotypes.

**Conclusions:**

Colonisation of the nasopharynx in Gambian infants by *S. pneumoniae *is rapid and highly dynamic. Immunity or inter-serotype competition may play a role in the dynamics. Reducing mother-infant transmission would have a minimal effect on infant carriage.

## Background

*Streptococcus pneumoniae *is a leading cause of invasive bacterial meningitis, acute respiratory infections and pneumonia among infants and children less than two years in developing countries [1]. Approximately 2.6 million preschool children die from pneumonia annually in developing countries, and almost half of these deaths are attributable to *S. pneumoniae *either exclusively or in conjunction with viral infections and/or malnutrition [2-5].

Nasopharyngeal carriage of *S. pneumoniae *is a risk factor for the development of respiratory diseases and pneumonia [6-8]. Although the relationship between carriage and disease is not well understood, evidence suggests that local or systemic invasive infection is caused by serotypes that bind to the epithelial surface of the respiratory tract [9-11]. Nasopharyngeal carriage is also the source of pneumococcal spread to other individuals mainly in intimate and overcrowded conditions. For example day-care centers are notorious for *S. pneumoniae *carriage and transmission among both day-care attendees and their siblings [12,13]. Over 80% of apparently healthy Gambian infants are nasopharyngeal pneumococcal carriers [14,15]. Carriage is also relatively high (50%) among Gambian adults [15].

*S. pneumoniae *is the most common cause of invasive bacterial disease (IBD) among Gambian children [16-18], with the highest rates occurring in infancy. Early diagnosis and treatment with antibiotics has been a successful strategy of managing cases but the emergence of drug resistant serotypes is making this strategy increasingly less effective and costly [19,20]. The search for a vaccine that would induce strong immunity against *S. pneumoniae *infection is therefore essential.

A randomised controlled trial of a 9-valent pneumococcal conjugate vaccine (PCV-9) among Gambian children was 77% efficacious against invasive pneumococcal disease caused by vaccine serotypes, 50% against disease caused by all serotypes, and 15% and 16% against all-cause admissions and mortality respectively [21]. Introduction of the same vaccine among day-care attendees in Israel reduced carriage of the vaccine serotypes, which are most associated with antibiotic resistance among recipients [22]. It is therefore very likely that introducing a higher valent PCV would be more effective in controlling both nasopharyngeal carriage and IPD. However, it is important to understand the distribution and dynamics of the carriage of the serotypes including those covered and those not covered by such a vaccine.

The collection of nasopharyngeal swabs from 196 mother/infant pairs during a high-dose vitamin A study conducted in six rural Gambian villages [14] offered an opportunity for us to study the longitudinal distribution and dynamics of pneumococcal carriage. We employed novel statistical techniques to describe the transmission and dynamics of carriage.

## Methods

The study described here formed part of a randomised placebo-controlled early-high-dose vitamin A supplementation trial, which was set up to test whether the new International Vitamin A consultative Group's recommended early high-dose regime of vitamin A supplementation would increase maternal and infant plasma vitamin A, reduce infant *Helicobacter pylori *infections and nasopharyngeal pneumococcal carriage and improve infant gut epithelial integrity. The study was conducted in six villages in the West Kiang region of The Gambia. Detailed description of the study area and population, including the methods of subject randomisation, recruitment and follow-up were given elsewhere [14]; only the methods used in the pneumococcal carriage component will be briefly discussed here. A total of 196 mother-infant pairs from the six villages were included in this study. Subjects were recruited from birth after completion of the appropriate consent procedures. The study protocol was explained to the subjects and their consent sought and confirmed by a formal signature/thumb print of the consent form. Subjects were followed for 12 months.

### Sample collection

Trained field assistants collected nasopharyngeal swabs from mother-infant pairs at birth (within 12 hrs of delivery), 2, 5 and 12 months using a sterile paediatric calcium alginate swab with flexible aluminium shaft (Fisher brand, Fisher Scientific UK Ltd, Loughborough, Leicestershire). All samples were collected in the clinic during the "study days" except those at birth which were collected in the subject's home or where she delivered. The swabs were immediately inoculated into 1 ml vials of skim milk tryptone-glucose-glycerin transport medium and brought to the laboratory in cold boxes within two hours of collection. The vials were then vortexed for 15 sec and then stored at -70°C until transported to the laboratory at MRC Fajara for further processing.

### Laboratory analysis

Standard techniques for culturing, isolation and serotyping were used to identify pneumococcal serotypes [23]. The frozen specimens were left to fully thaw at room temperature and then mixed thoroughly before being streaked onto selective agar plates of 5 mg/L gentamicin-trypticase soy agar with 5% sheep blood. The plates were then incubated overnight at 37°C in 5% CO_2_. Phenotypic characteristic of morphological analysis and α-haemolysis were used for presumptive identification of pneumococci colonies. Three to four colonies with typical pneumococci morphology were selected and pneumococci confirmed by Optochin susceptibility and bile solubility test. The Optochin test involved sub-culturing of the colonies by streaking out each of the selected colonies on one half of two of the selective agar plates and placing an optochin disc in the centre of the streaks. The plates were then incubated overnight as before. A positive culture of S. pneumoniae with optochin disc had an inhibition zone of at least 14 mm around the disc and a negative culture had no inhibition zone. A single positive colony was then selected from each of the streaks and serotyped by Quellung reaction. Serotypes were categorised under vaccine and non-vaccine types based on the 13 valent vaccine which consists of serotypes 1, 3, 4, 5, 6A, 6B, 7F, 9V, 14, 18C, 19A, 19F and 23F for data analysis.

#### Ethical approval

The study was approved by the MRC Scientific Coordinating Committee, the joint MRC and Gambia Government Ethics Committee and the Ethics Committee of the London School of Hygiene and Tropical Medicine, UK.

#### Statistical Methods

Tabulation, Pearson χ^2 ^tests and logit- and log-linked binomial regression were performed with Stata version 9 (StatCorp LP, TX, USA).

Serotype data from the four time points were considered in separate and pooled logistic regression analyses, and also combined as a binary "ever detected" variable, in which individuals scored one if the serotype was detect at one or more time point, zero if not. Whenever the presence or absence of serotype was the dependent variable (i.e. analyses of age, sex and seasonality and the estimation of OR in the calculation of PAF, but not the regression used to estimate half-life - see below) the data were analysed with logistic regression. In all these logistic regression models, serotype differences were fitted using the single term logit(serotype prevalence). This was used because more conventional models fitting serotype as a fixed categorical factor or a random effect were unstable, sometimes failing to converge (probably because the approximations made by these models break down when the prevalence of many serotypes is very low).

In all regression analyses (now including both the logistic and log-linked binomial regression) in which time points were pooled we applied robust (Huber-White) standard errors to account for between-individual variation. This was preferred to the less stable multi-level models with individual modelled as a random effect.

Seasonality was fitted using a truncated Fourier series [24]. Briefly, in this case, the first two pairs of Fourier terms (sinθ, cosθ and sin2θ, cos2θ, where θ is the angle in radians representing the point in the annual cycle at which the observation was made) were entered as independent variables in logistic regression models.

We estimated the contribution of mother-to-child transmission to infant carriage by considering the increased risk of an infant carrying a particular serotype if the mother carries the same serotype. This requires a slight modification of the standard population attributable fraction (PAF) calculation in order to accommodate stratification by serotype. We first calculate the PAF for each serotype using the standard formula:

Where:

P_e _= proportion of children exposed, i.e. serotype prevalence among mothers,

AF = attributable fraction = (RR-1)/RR,

RR = risk ratio (risk if exposed/risk if not exposed) = I_e _/I_u_

I_u _= serotype prevalence among unexposed infants,

I_e _= serotype prevalence among exposed infants,.

P_e _is readily calculated for each serotype but RR, and hence AF, estimates for serotypes with low prevalence are very inaccurate and unreliable. Instead we obtain a common estimate of OR for all serotypes from logistic regression and used the relationship AF = (1-I_u_).(OR-1)/OR to calculate the serotype-specific AF. A score reflecting the overall fraction of infant pneumococcal carriage attributable to maternal carriage was then obtained by taking a weighted average of the serotype-specific PAFs with weights equal to the serotype prevalence among infants. The 95% confidence interval we give for the overall PAF was based on the precision of the OR estimate. (Note, this does not take into account imprecision in the estimation of the serotype prevalences; the confidence interval gives the precision of our estimate of the PAF for a population with serotype prevalence structure exactly as we estimate. This should provide a reasonably representative figure. Making allowance for imprecision in the prevalence estimation would be relevant only for the particular situation in Kiang West at the time of the study).

A permutation test was devised to compare the distribution of the observed number of serotypes per individual with that predicted assuming that the serotypes were distributed independently. To do this we first simulated the carriage of each serotype for each of 100,000 individuals such that the probability that a particular individual "carried" a particular serotype was equal to its observed prevalence at the time point of interest (or, in the case of the "ever-carried" variable, the proportion of individuals in which it was observed). Thus the assignment of a serotype to an individual was made independently of which other serotypes the individual carried and of the carriage of other individuals. Next, counting how many serotypes each "carried", we calculated the expected proportions of individuals carrying 0, 1, 2, etc serotypes and multiplying these by 196 we obtained the corresponding expected frequency in our sample. Then we used Pearson's χ^2^-test to compare this simulated distribution with the observed frequencies. We also calculated the ratio of the observed to expected variance and estimated its 95% confidence interval by assuming that the sum of squares divided by the expected variance should approximately follow a χ^2^-distribution under the null hypothesis. (We note that the bootstrap yielded an almost identical confidence interval.) This analysis was carried out on an MS-Excel worksheet.

In order to calculate the half-life of pneumococcal colonies in infants and their mothers we first defined a variable indicating, for each serotype, individual and time point (other than birth), whether the serotype had been lost since the previous time point. If the serotype had not been isolated at the previous time point the variable was coded unknown. This variable was regressed on the length of the interval (in months) between the current and previous time points using log-linked binomial regression with no constant and the rate of colony loss estimated from the regression coefficient, β: colony half-life = log(0.5)/β.

Difference in dynamics between serotypes is of considerable interest. However, individual serotype prevalences are mostly very low, so fixed or random effects models were deemed impractical. Instead we concentrated on the broad differences between vaccine and non-vaccine serotypes.

## Results

### Serotype distribution

A total of 1568 nasopharyngeal swabs were taken from all 196 mothers and their infants at each of four time points. These yielded 741 isolates and a total of 46 identified serotypes. Table 1 shows the distribution of serotype prevalence. Ninety eight percent of children carried pneumococci at some point during the study and all 13-valent pneumococcal conjugate vaccine serotypes (Table 1) were isolated from both infants and mothers.

**Table 1:** Distribution of prevalence of vaccine and non-vaccine serotypes listed in order of prevalence among infants

	Infants					Mothers				
***non-vaccine*** ***serotypes:***	Infant's age (months)									
	0	2	5	12	ever*	0	2	5	12	ever*
18B					0.0%	0.5%				0.5%
39					0.0%	0.5%				0.5%
48					0.0%			0.5%		0.5%
18A					0.0%			0.5%	0.5%	1.0%
35F					0.0%			0.5%	0.5%	1.0%
35C			0.5%		0.5%					0.0%
47		0.5%			0.5%					0.0%
24			0.5%		0.5%	0.5%				0.5%
9N				0.5%	0.5%				0.5%	0.5%
19C				0.5%	0.5%		0.5%	0.5%		1.0%
20		1.0%	0.5%		1.0%				0.5%	0.5%
2		0.5%		0.5%	1.0%		0.5%	0.5%	0.5%	1.5%
23A		1.0%	0.5%		1.5%		0.5%			0.5%
31		1.0%	0.5%		1.5%				0.5%	0.5%
16		1.0%	1.0%	0.5%	2.0%				0.5%	0.5%
38			0.5%	1.5%	2.0%		0.5%	0.5%		1.0%
17		0.5%	1.5%		2.0%		1.0%	0.5%	1.0%	2.0%
28		1.5%	1.0%		2.0%		1.0%	1.0%		2.0%
22		2.0%	0.5%		2.6%			0.5%	0.5%	1.0%
9L		0.5%	1.5%	1.0%	2.6%	0.5%		0.5%		1.0%
23B		1.5%	1.5%	0.5%	3.1%	1.0%	1.0%	1.0%	1.0%	3.6%
33		2.0%	2.0%		3.6%		1.0%			1.0%
10		3.1%	1.5%		3.6%	1.0%	1.0%	0.5%		2.0%
12		2.6%	2.0%		4.1%					0.0%
7C		3.1%	3.1%		4.1%		1.0%	0.5%		1.5%
19B		1.5%	2.6%	1.5%	4.6%	0.5%	1.0%	0.5%	0.5%	2.6%
9A			4.6%	1.0%	5.6%			1.0%		1.0%
13		2.0%	1.0%	2.6%	5.6%		1.0%		0.5%	1.5%
21		2.6%	2.6%	1.0%	5.6%	1.5%		0.5%		2.0%
11		2.6%	2.0%	2.0%	6.1%	1.5%	1.0%	0.5%		3.1%
35B		2.6%	2.6%	2.0%	6.1%		1.0%	0.5%	2.0%	3.1%
34	0.5%	4.6%	0.5%	1.0%	6.1%	2.6%	1.5%	1.5%		5.1%
15		7.1%	6.1%	6.1%	15.3%		2.0%	0.5%	1.0%	3.6%
any non-vacc	0.5%	43.4%	41.3%	23.0%	73.0%	10.2%	16.8%	13.3%	9.7%	38.8%

***13-PCV serotypes**:*										
7F			0.5%		0.5%			0.5%		0.5%
1			0.5%	0.5%	1.0%					0.0%
5			1.0%		1.0%					0.0%
18C †		1.0%		0.5%	1.5%		1.0%		1.0%	2.0%
3		1.0%	1.0%	0.5%	2.6%		0.5%	0.5%	0.5%	1.5%
9V †		0.5%		3.1%	3.6%		0.5%		1.0%	1.5%
4 †		2.6%	1.0%	1.0%	4.6%	0.5%	0.5%		0.5%	1.5%
19A		1.5%	5.6%	5.6%	11.2%	0.5%	0.5%	2.6%	0.5%	3.6%
23F †		8.2%	7.1%	6.6%	17.3%	0.5%	0.5%	1.0%	2.6%	4.6%
14 †		4.1%	7.7%	11.2%	20.9%	0.5%	2.0%	1.0%	1.5%	4.1%
6B †		8.7%	15.3%	12.2%	26.5%	1.0%	0.5%	2.0%	1.0%	4.6%
6A		6.6%	12.8%	12.8%	27.0%		0.5%	2.0%	2.6%	5.1%
19F †	1.0%	7.1%	8.2%	15.8%	27.6%	0.5%	0.5%	2.0%	1.0%	4.1%

any vacc	1.0%	39.3%	58.2%	63.3%	89.3%	3.1%	7.7%	11.2%	12.2%	28.6%

any serotype	1.5%	78.1%	87.8%	78.6%	98.0%	13.3%	24.5%	23.5%	21.4%	55.1%

* "ever" here refers to the detection of the serotype at any one of the four time points

† component of 7-valent vaccine

(N = 196 throughout).

### Infant's age and sex

**Figure 1:**
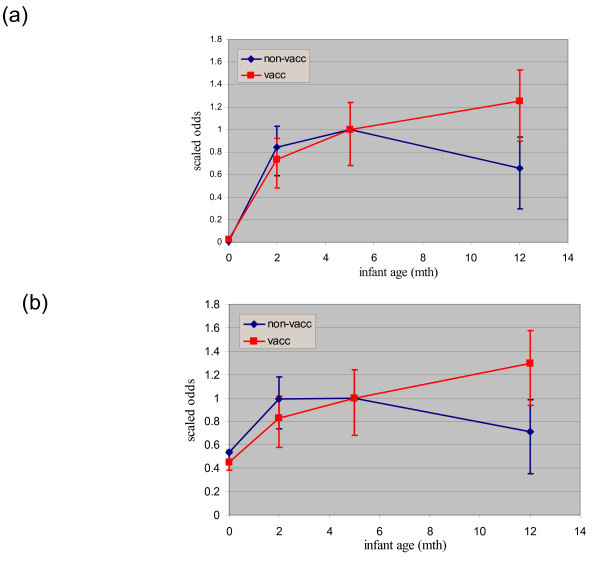
**(a) & (b) - Graph of the odds of carriage between vaccine and non-vaccine serotypes among infants and mothers**. Pink line = vaccine serotypes; Blue line = non-vaccine serotypes. The odds on the y-axis have been scaled (by dividing by the value at 5 m) in order to facilitate visual comparison of the patterns.

The percentage of infants carrying at least one serotype increased rapidly from 1.5% at birth to 77% at 2 months of age, remaining fairly constant thereafter. Maternal carriage doubled over the same interval and remained elevated thereafter (Table 1). There is some evidence that this pattern differed between serotypes: logistic regression demonstrated a significant interaction between time point (i.e. infant's age) and whether the serotype was a vaccine or non-vaccine serotype (X^2 ^= 14.3 on 3 df; p = 0.0025): while vaccine serotypes continued to increase in prevalence with infant age, the non-vaccine serotypes reached a peak and by one year had fallen well below their levels at five months (Fig 1). A similar pattern was seen among mothers, although the prevalences were lower, the power weak and the interaction not significant.

The inclusion of a term for infant's sex did not significantly improve these models and was excluded from subsequent analysis.

### Seasonality

**Figure 2:**
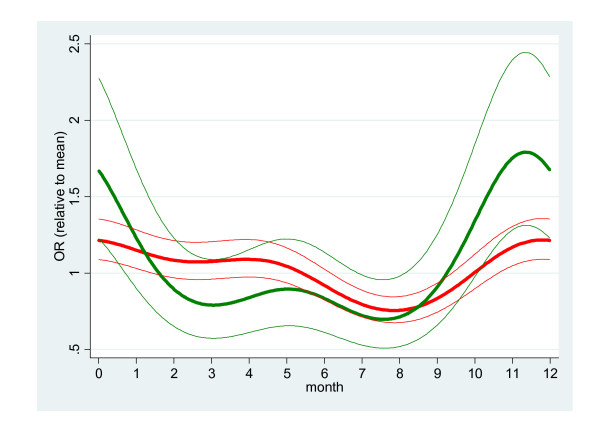
**Seasonality of carriage among mothers and infants**. Red = infant; green = mother. Thick = best fit; thin = 95% confidence limits.

The pattern of carriage for both infants and mothers was significantly seasonal: in a logistic regression model controlling for serotype prevalence and time point (infant's age) the first two pairs of Fourier terms were found to be (jointly) significant (children: X^2 ^= 25.4 on 4 df, p < 0.0001; mothers: X^2 ^= 16.9 on 4 df, p = 0.002). While the pattern appears to be more exaggerated for mothers (Fig 2) fitting separate patterns for mothers and children was not significantly better than a common pattern (X^2 ^= 7.54 on 4 df, p = 0.11). Similarly, there was no evidence to suggest that the vaccine and non-vaccine serotypes exhibit different seasonality among children (X^2 ^= 8.00 on 4 df, p = 0.092).

### Distribution of the number of serotypes per individual

**Figure 3:**
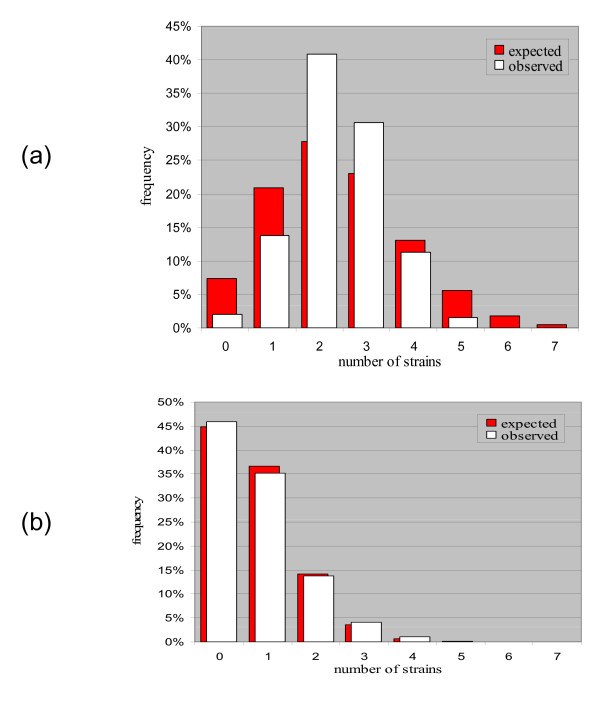
**(a) & (b) - Distribution of the observed and the expected number of serotypes each individual was found to be carrying at one or more time point**. Red bar = expected. White bar = observed

The distribution of the number of serotypes ever carried by infants (i.e. at any of the four time points) differed significantly from what would be expected if the serotypes were assorted independently (X^2 ^= 40.1 on 7 df; p = 1×10^-6^. Fig 3(a)). This was due to considerable under-dispersion: the variance was 45% of that expected (CI_95%_: 39% - 58%).

Curiously, there was no evidence that the distribution of the number of serotypes carried by mothers was under-dispersed (Fig 3(b)): the variance among mothers was 105% of that expected and the 95% confidence interval, 87% - 130%.

### Rates of loss of serotypes

The estimated half-life of pneumococcal colonies in infants, 1.90 m (CI_95%_: 1.66 - 2.21), was significantly longer than that in mothers, 0.75 m (CI_95%_: 0.55 - 1.19). Furthermore, vaccine serotypes were, on average, retained considerably longer than non-vaccine serotypes in both infants: 2.24 m (CI_95%_: 1.89 - 2.75) vs 1.51 m (CI_95%_: 1.25 - 1.91), p = 0.008. A similar pattern is seen for mothers: 1.03 m (CI_95%_: 0.64 - 2.62) vs 0.58 m (CI_95%_: 0.43 - 0.91) although this difference was not significant. Including either a constant or quadratic term in these models provided no improvement in fit so we have no evidence to contradict the assumed exponential decay model or to suggest that loss was age-dependent (age and interval length are strongly confounded in this study design).

### Relationship between mother's and infant's carriage

**Figure 4:**
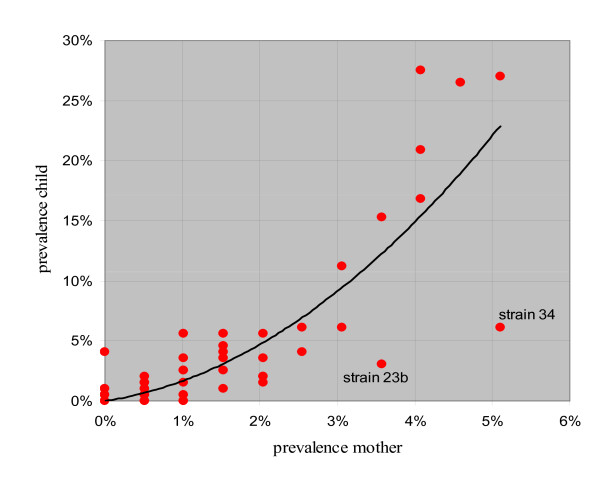
**Relationship between serotype prevalence in mothers and infants**.

It can be seen from Fig 4 that there is a close relationship between the prevalence of a serotype in infants and its prevalence in mothers, two exceptions being serotypes 23b and 34 (for the latter it is highly unlikely to be due to chance) both of which are less prevalent in infants than expected. Logistic regression shows that the probability that an infant ever carried a particular serotype is much increased if their mother ever carried it: OR = 9.1 (CI_95%_: 6.4 to 13.6). While this value is comparatively high, the prevalence of most serotypes is low and maternal carriage accounts for a small percentage of infant carriage: PAF = 9.5% (CI_95%_: 7.4 to 11.6%) in the "ever carried" analysis.

There was no evidence that this relationship varied with age (X^2 ^= 3.37 on 2 df; ns). The PAFs estimated separately at 2 m was slightly higher than later time points but the tight and overlapping 95% confidence intervals rule out anything but a modest age dependence (at 2 m PAF = 10.5%, CI_95%_: 7.1 to 13.5%; at 5 m PAF = 7.3%, CI_95%_: 4.5 to 10.1; at 12 m PAF = 7.2%, CI_95%_: 4.6 to 9.7%).

The OR is significantly smaller for vaccine serotypes than others (OR for vaccine serotypes = 5.4, CI_95%_: 3.1 to 9.6; OR for non-vaccine serotypes = 14.1, CI_95%_: 8.4 to 23.5; p = 0.018). These translate into a PAF due to maternal carriage of 7.0% (CI_95%_: 4.4 to 9.6%) for vaccine serotypes and 13.3 (CI_95%_: 9.5 to 17.3%) for non-vaccine serotypes.

These associations include a possible contribution due to infants infecting their mothers. To eliminate this it is tempting to consider the association between infant carriage at a particular time point with maternal carriage at the previous time point. However, these correlations were found to be very weak, probably due to the very short half-life of colonies in infants and, especially their mothers.

## Discussion

While there has been a previous longitudinal study of nasopharyngeal carriage of *S. pneumoniae *among Gambian infants [25] ours is the first to study *S. pneumoniae *carriage simultaneously among mother-infant pairs in the Gambia. Our results are consistent with the findings of a similar study conducted among mother infant pairs in Papua new Guinea [26]. Our findings provide baseline information on the dynamics of carriage that we hope will prove useful in the preparation for the introduction and evaluation of the 13-valent conjugate pneumococcal carriage in The Gambia and, more generally, in understanding the transmission of the infection in infancy.

The data we report here was obtained from a vitamin A intervention trial, the results of which were published earlier [14]. The intervention had no detectable effect on pneumococcal carriage and its inclusion in the analyses of transmission had a negligible effect. We have therefore ignored it in the results we present here.

We have isolated 46 serotypes (serotypes), identified the most common serotypes of both vaccine and non-vaccine serotypes and demonstrated that some Gambian infants already carry nasopharyngeal pneumococci within 12 hours of delivery and have reached a steady level of approximately 80% by 2 months of age. This is consistent with the findings of similar studies carried out elsewhere [27,28] and in The Gambia by Hill and colleagues [15]. Hill *et al *have demonstrated nasopharyngeal pneumococcal colonization in Gambian infants as early as at 7 days postpartum and showed that the mean and median ages of first acquisition of carriage were 33 and 24 days respectively [25].

In several nasopharyngeal carriage studies, only few serotypes form the most common serotypes [27,29]. In our study, 67% of the serotypes ever carried by the infants are the 6 most common serotypes identified among infants. Four of these are vaccine serotypes included in the 7-valent conjugate vaccine (serotypes 4, 6B, 9V, 14, 18C 19F and 23F) forming 92.3% of the vaccine serotypes ever carried by the infants. In most countries where immunization with the 7-valent vaccine has been successful, over 60% of serotypes have been covered by the vaccine [30-34]. Only 43% of serotypes carried by infants in our study are covered by the 7-valent conjugate vaccine, a similar figure to that reported for infants by Hill et al [25]. This is of relevance for the introduction and evaluation of the 13-valent conjugate vaccine, although the picture is confused by results from an earlier study reporting a coverage of 63% of isolates among Gambian under-5 year olds [15].

Counting the number of serotypes each infant was observed to have carried at one or more time points gives a distribution that is considerably, and highly significantly, under-dispersed relative to simulations under the assumption of independent assortment of serotypes would lead us to expect. There is a strong tendency for count distributions in nature to be over-dispersed due to clustering [35]. Under-dispersion is a less commonly encountered phenomenon and implies that there is some sort of ordering in the system strong enough to cancel out the expected clustering. Possible reasons for such ordering in this system include cross-immunity between serotypes, active competition between serotypes or technical issues associated with the laboratory processing of the samples. The last of these is of particular concern for the Optochin method employed here. Before concluding that the observed under-dispersion reflected a biological phenomenon it would be important to repeat this analysis on serotype data derived from a PCR-based method. Among our mothers there was little evidence that serotypes were not independently assorted and clearly the confidence interval for the ratio of the observed to the expected variance indicates that levels of under-dispersion similar to that seen among infants would be highly implausible. One explanation might be that whatever causes the under-dispersion among infants is a non-linear process, disproportionately weaker when the average number of serotypes carried is lower. Another explanation might be that mothers' immune systems are already fully primed with respect to these bacteria; unlike their immunologically inexperienced infants, seeing more serotypes do not further change their immunological status. Some support for this idea is lent by the considerably shorter lifespan of colonies in mothers than infants. Mothers in our study carried fewer serotypes on average than children. This is consistent with a study carried out among mother-infant pairs from birth in Papua New Guinea, to determine the age of acquisition and duration of carriage of the first strains of *Haemophilus influenzae *and Streptococcus pneumoniae in the upper respiratory tract [26]. The study found that mothers, generally either did not carry *H. influenzae *or *S. pneumoniae*, or harboured types different to those first acquired by their infants. However, one-third of mothers subsequently became colonized with *H. influenzae *and *S. pneumoniae *types similar to those carried by their babies.

Previous pneumococcal carriage has been found to be associated with serotype-independent protection from subsequent acquisition, whereas recent serotype-specific exposure within the family has been found to be associated with an 8-fold increase in the rate of acquisition of that serotype [36]. This is found to be consistent with the hypothesis that serotype-independent protective immunity is activated in children by previous carriage and reduces the rate of new colonisation. Cross immunity in the system is also consistent with the principle of herd immunity [37]. Dagan and colleagues have demonstrated in toddlers an inverse relationship between pneumococcal-conjugate vaccine (PCV) post-vaccination high serum concentration of serotype-specific pneumococcal anticapsular IgG and reduced probability of new acquisition of carriage [7], suggesting that the magnitude of herd protection against S. pneumoniae provided by PCV may depend on the magnitude of IgG concentration. However, a review by Lipsitch *et al *suggests that anticapsular antibody is not primarily responsible for the age-specific decline in invasive pneumococcal disease, that other factors such as age-related anatomical changes and innate or acquired cellular immune response cannot be ruled out and that more than one factor may be involved [38].

Not only is colonisation of infants by pneumococcus rapid, it appears to be a highly dynamic process. The half-life of a serotype in infants is only 1.9 months and 0.75 months in mothers. (Even these estimates may be inflated by the loss and subsequent recolonisation of serotypes during the long interval between swabs. On the other hand under-estimation of the half-life would result from less than perfect sensitivity of the detection method.) After 2 months of age the proportion of children carrying at least one serotype has reached 77% and remains at approximately 80% until 12 months of age, although the serotypes carried at 2 months are rapidly replaced by others. This is consistent with the literature[39,40] and, given the epidemiology of pneumococcal nasopharyngeal carriage in this age group [15,16,25], not surprising. A study in the Gambia found a 79% prevalence of carriage of non-vaccine serotypes among children vaccinated with 3 doses of pneumococcal conjugate vaccine, compared to a 42.5% prevalence among control children [41]. Active competition between serotypes has been observed in populations after the introduction of the 7-valent conjugate vaccine [39,40,42] when non-vaccine serotypes replace vaccine serotypes in a process known as serotype replacement. Carriage of non-vaccine serotypes increased among children receiving the conjugate vaccine to a level where the overall prevalence of pneumococcal carriage has not been different in vaccinated and unvaccinated children [37]. Another study in South Africa also found evidence of serotype replacement in a 9-valent vaccine trial among children where the prevalence of non-vaccine serotypes increased from 21% in the controls to 39% in the vaccinated [43]. Nonetheless, it must be noted that the prevalence of carriage changes much more gradually than the incidence of invasive disease [44]. However, serotype replacement is a crucial phenomenon in the use of vaccination to prevent pneumococcal disease; its role, dynamics and implications for the development and widespread use of vaccines for the control of pneumococcal disease requires further investigations. The rapid turnover of serotypes in our study suggests that some serotypes may be more rapid colonisers than others. Our data provide some support for this notion: in Fig 2 we see that the proportion of vaccine serotypes increases as the infant ages. It is difficult to determine whether colony half-life changes with infant's age because the length of the interval between time points is strongly correlated with age: the half-life/age interaction is heavily confounded with the possibility that the hazard of serotype loss is not uniform with respect to the colony's age.

Although carriage is seasonal among both mothers and infants, there is no evidence to suggest different seasonality of carriage between vaccine and non-vaccine serotypes. The higher carriage rates seen in the dry season may explain the seasonality of invasive pneumococcal disease among children in the Gambia [18]. However, it is interesting to note that the two serotypes, 1 and 5 that form over a third of serotypes causing invasive pneumococcal disease in the Gambia are rarely carried [15,45].

We estimate that, while an infant is 9 times as likely to carry a particular serotype if their mother does, the population attributable fraction due to maternal carriage is less than 10%, and this figure is probably inflated both by infant-to-mother transmission (a mother's carriage on average doubles in the first two months of her infant's life) and simultaneous transmission to both from a third party. We could not determine the extent of these because of our study's key weakness was the infrequency of swabbing and the extended time between swabs. We have not extended our study to other members of the family for example siblings and caregivers. These weaknesses were all as a result of the fact that the study was not originally designed to describe the dynamics of carriage among this cohort.

Much of the analysis applied here relies on the assumption that the pneumococcal serotypes are homogeneous. While we appreciate that this will not always be true (indeed, we demonstrate that vaccine and non-vaccine serotypes do often differ) nevertheless we feel there is value in estimating the average patterns and parameters that this approach yields. The prevalence of most is too low to make such estimates for individual serotypes. We note, however, that if the lifespan of colonies were heterogeneous the marginal distribution of survival times would no longer be exponential and estimates of half-life less meaningful.

## Conclusions

Nasopharyngeal pneumococcal carriage is highly prevalent among mother-infant pairs. Colonisation of the nasopharyngeal tract in Gambian infants by *S. pneumoniae *is rapid and highly dynamic. Equilibrium between acquisition and loss of colonies is reached by 2 m of age and their half-life is only 1.9 m. The under-dispersed distribution of the number of serotypes carried by an infant suggests that immunity or inter-serotype competition may play a role in the dynamics. While there is a strong association between maternal and infant serotype carriage, more than 90% of infant carriage is derived from sources other than the mother. There is evidence that the half-life of colonies, age-dependence of carriage and relative contribution of mother-infant transmission differ markedly between the vaccine and non-vaccine serotypes.

## Competing interests

The authors declare that they have no competing interests.

## Authors' contributions

MKD participated in the design, carried out the field work and wrote the first draft. AJCF performed the statistical analysis and participated in the writing. OS participated in and supervised the laboratory analysis. AMP conceived the original study and provided advice and funding. All the authors have read and approved the manuscript.

## Pre-publication history

The pre-publication history for this paper can be accessed here:

http://www.biomedcentral.com/1471-2334/10/195/prepub
